# The Effects of Chemical Bonding at Subatomic Resolution: A Case Study on α-Boron

**DOI:** 10.3390/molecules26144270

**Published:** 2021-07-14

**Authors:** Andreas Fischer, Georg Eickerling, Wolfgang Scherer

**Affiliations:** Institut für Physik, Universität Augsburg, Universitätsstraße 1, D-86159 Augsburg, Germany; andreas.fischer@uni-a.de (A.F.); georg.eickerling@uni-a.de (G.E.)

**Keywords:** charge density, subatomic resolution, core asphericity shift, α-boron

## Abstract

Similar to classical *asphericity shifts*, aspherical deformations of the electron density in the atomic core region can result in *core asphericity shifts* in refinements using a Hansen-Coppens multipolar model (HCM), especially when highly precise experimental datasets with resolutions far beyond sin(θ)/λ ≤ 1.0 Å^−1^ are employed. These shifts are about two orders of magnitude smaller than their counterparts caused by valence shell deformations, and their underlying deformations are mainly of dipolar character for 1st row atoms. Here, we analyze the resolution dependence of core asphericity shifts in α-boron. Based on theoretical structure factors, an appropriate Extended HCM (EHCM) is developed, which is tested against experimental high-resolution (sin(*θ*)/*λ* ≤ 1.6 Å^−1^) single-crystal diffraction data. Bond length deviations due to core asphericity shifts of α-boron in the order of 4–6·10^−4^ Å are small but significant at this resolution and can be effectively compensated by an EHCM, although the correlation of the additional model parameters with positional parameters prevented a free refinement of all core model parameters. For high quality, high resolution data, a proper treatment with an EHCM or other equivalent methods is therefore highly recommended.

## 1. Introduction

The refinement of X-ray structure factors with an independent atom model (IAM) causes systematic bond length errors, so-called *asphericity shifts*, due to the aspherical nature of the valence shell of covalently bonded atoms in molecules [1,2,3,4,5]. These differences become obvious when structure models based on X-ray data (X) and neutron data (N) are compared. Similar comparisons can also be made when the same X-ray dataset is refined using both an IAM and a Hansen-Coppens multipolar model (HCM) [6], where the latter takes the aspherical valence density into account. In IAM refinements the deformation of the valence shell due to chemical bonds or lone pairs is partially compensated by a small displacement of the respective atom [5,7]. These errors can become relatively large for light atoms, where the valence density dominates the total electron density. Accordingly, the largest bond length differences of 0.096(7) Å and 0.155(10) Å can be observed in X-N comparisons in case of C-H bonds and O-H bonds, respectively [5]. However, C-C bonds are also affected by asphericity shifts, especially when multiple bonds like the C≡C triple bond of the acetylene moiety in the organometallic complex [Ag(η^2^-C_2_H_2_)]^+^[Al(OC(CH_3_)(CF_3_)_2_)_4_]^−^ are involved, which appears about 0.063(5) Å shorter in the IAM compared to the HCM [8,9].

**Figure 1:**
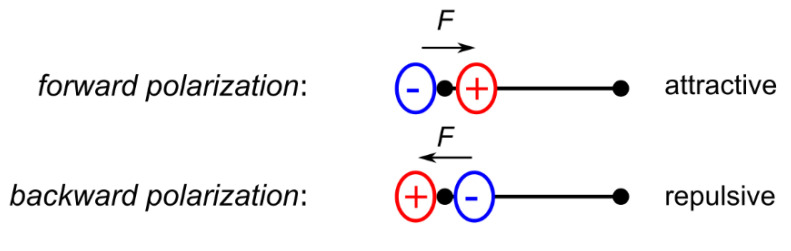
Two cases of dipolar core-polarization of first-row atoms.

Due to the nodal structure of atomic orbitals, electron density deformations in the valence region are also noticeable in the core region and might result in complex polarization effects when transition metals are investigated [10]. According to Bentley and Stewart, core-deformations of first-row atoms are mainly of dipolar nature [11,12,13]. This has later been confirmed by Chandler and Spackman for diatomic hydrides *A*H (*A* = B, C, N, O) [14]. These dipolar polarizations are sharply localized around the nucleus and generate an electrostatic Hellman–Feynman force at the nucleus, which can be, depending on the orientation of the dipole (Figure 1), attractive or repulsive with respect to the bonding electron density. At a stationary point, it precisely compensates the respective force generated by the aspherical valence electron density distribution [15,16,17]. Although the magnitude of the electron density contribution of these dipoles is small compared to the total electron density, they result in quite high electric fields due to the small distances to the nucleus [17,18].

Dipolar core polarization also leads to a shift of the atomic position at the HCM level. It is about two orders of magnitude smaller than classical asphericity shifts and becomes observable and significant only at high data resolutions beyond sin(θ)/λ ≤ 1.0 Å^−1^, where atomic positions and bond distances can be determined to a sufficient accuracy. Hirshfeld observed shifts of 0.0006(3) Å for carbon atoms and 0.0007(2) Å for fluorine atoms in 1,4-dicyano-2,3,5,6-tetrafluorobenzene when dipolar core polarization is taken into account, for example by using a Hellman–Feynman constraint [19]. Furthermore, he showed that dipolar core polarization is highly correlated with the atomic position and suggested that it is unlikely to achieve an independent refinement, even at significantly increased resolution and/or lower temperatures. An alternative is the precise determination of atomic positions from single crystal neutron diffraction experiments with a precision of around 0.0001 Å [19,20]. To avoid confusion with classic asphericity shifts, shifts due to core polarization effects are termed *core asphericity shifts* in the following.

Due to the frozen core assumption, core polarization effects cannot be refined with the standard HCM (for details and equations, see Appendix A). This assumption breaks down for high resolution data sets, and it has been shown in case of the diamond modification of elemental carbon that an extension of the HCM (EHCM) is necessary to avoid systematic errors of the atomic displacement parameters [21,22,23]. For diamond, the introduction of a core expansion/contraction parameter *κ*_c_, in combination with a possible charge-transfer between the core (*P*_c_) and valence (*P*_v_) pseudo atom and radial density functions of a carbon atom in its ^5^S state yielded not only satisfactory fits for theoretical, static structure factors but also the temperature factors extracted from experimental diffraction patterns (sin(*θ*)/*λ* ≤ 1.45 Å^−1^) recorded at a 3rd generation synchrotron source were in much better agreement with inelastic neutron scattering experiments [22]. In further studies with experimental resolutions up to sin(*θ*)/*λ* ≤ 1.7 Å^−1^ by Bindzus et al. and Svane et al. even a free refinement of the parameters *P*_c_ and *κ*_c_ was possible and in accord with the earlier theoretical predictions [24,25]. While the core pseudo atom in diamond shows only spherical deformations, significant aspherical core deformations are present in the case of the isostructural silicon phase, and a total of three pseudo atoms are necessary to describe the static electron density at resolutions up to sin(*θ*)/*λ* ≤ 1.8 Å^−1^ [22]. Using synchrotron powder diffraction, these aspherical deformations, however, could not yet be determined experimentally, but many other systems have been studied in the meantime using an EHCM [25,26,27,28,29]. Hence, the EHCM is the method of choice to gain precise structural parameters using high resolution single crystal data of extended solids [29,30]. If *d*- or *f*-electrons are involved, however, the corresponding EHCMs can become quite complex [10].

In addition to the EHCM, there have been further attempts to overcome the limits of the HCM. Koritsanszky et al. suggested to replace the *m*-independent single-ζ radial density functions (RDF) in the HCM by *m*-dependent multiple-ζ RDFs which can be obtained from ab-initio calculations [31,32,33]. However, this idea is not yet implemented in any multipolar refinement code.

A method which is recently becoming increasingly popular is the Hirshfeld atom refinement (HAR), with a special focus on determining positions and ADPs of hydrogen atoms at a precision comparable to single crystal neutron scattering [34,35,36]. As hydrogen atoms have no core shell, their positions cannot be determined by high resolution data since all the information, including that of the aspherical valence density, is contained only in the low resolution reflections [37]. HAR is an iterative approach and makes use of scattering factors of aspherical atoms extracted from DFT calculations and subsequent stockholder partitioning of the molecular electron density, according to Hirshfeld [34,38]. However, HAR is limited to molecular systems, had problems to deal with heavy atoms (4th and 5th period), occupational and positional disorder, and was lacking an appropriate extinction correction, which is often necessary to handle highly crystalline solid state compounds [36]. Part of these limitations (disorder phenomena, presence of heavy atoms, extinction) have been lifted recently with the availability of NoSpherA2, which is an HAR implementation in the Olex2 software package [39]. HAR has also been applied to the solid-state ionic compound CaF_2_ by a cluster approach, since periodic solid state calculations are not yet implemented [39].

For the covalently bonded framework of α-boron, the EHCM therefore remains the best available approach to deal with core polarization effects which we therefore used throughout this study. It is commonly accepted that three types of chemical bonds are occurring in α-boron: localized intericosahedral (2c,2e) and (3c,2e) bonds, as well as delocalized intraicosahedral bonds [40]. The first experimental electron density studies of α-boron were performed by Will and Kiefer in 1987 and 2001 [41,42] based on a dataset by Morosin et al. [43]. The data were acquired at room temperature employing single crystals grown by F. H. Horn [44]. This dataset was limited to an experimental resolution of sin(*θ*)/*λ* ≤ 1.04 Å^−1^ and refined using the high-order low-order technique (HO-LO) and an HCM. The model was validated only by visual inspection of the residual density and dynamic deformation density maps while an analysis of the topology of the electron density was not performed.

A recent charge density study of α-boron by Mondal et al. using single crystals at *T* = 100 K uses synchrotron radiation with a maximum resolution of sin(*θ*)/*λ* ≤ 1.221 Å^−1^ [45]. The electron density distribution was analyzed within the framework of Bader’s quantum theory of atoms in molecules (QTAIM) and shows only a small charge transfer of 0.07 electrons between the two independent boron atoms of the asymmetric unit. Density values *ρ*(**r**_c_) and *L*(**r**_c_) values are reported for several selected bond-critical points (BCP) and ring-critical points (RCP). The new findings were at first in contrast to two earlier independent charge density studies based on synchrotron powder data (sin(*θ*)/*λ* ≤ 0.75 Å^−1^) evaluated by the maximum entropy method (MEM), which suggested a strongly bent nature of the intericosahedral (2c,2e) bond [46,47,48]. However, it was shown by Nishibori et al. in a synchrotron powder experiment conducted at the beamline BL44B2 (SPring-8, Japan) with resolutions up to sin(*θ*)/*λ* ≤ 1.529 Å^−1^ at *T* = 100 K that the earlier powder diffraction results were biased by systematic errors which disappear at resolutions above sin(*θ*)/*λ* ≤ 1.25 Å^−1^ when the MEM technique is used [49].

Despite the importance of the structural model of α-boron in main group chemistry, theoretical studies of α-boron employing a QTAIM analysis of the electron density are rare. Most studies are employing plane wave calculations using pseudo potentials and are limited to simple contour maps [50,51,52]. V. Sagawe analyzed the electron density distribution using LMTO and FP-LAPW calculations; however, the subsequent QTAIM analysis revealed non-nuclear attractors and missing critical points [53].

The aim of this study is therefore manifold. First, we provide high quality theoretical DFT calculations of α-boron employing the FP-LAPW technique and the full analysis of the electron density topology with QTAIM methods, which serve as a reference. Second, we have grown α-boron crystals of suitable size and quality from platinum flux at high pressures to conduct an experimental charge density study at high resolutions (sin(*θ*_max_)/*λ* = 1.579 Å^−1^). This dataset in combination with theoretical structure factors will be used to study the resolution dependence of *core asphericity shifts* which originate from dipolar core polarizations. Finally, we will show that an EHCM employing an aspherical modelling of the electron density in the core shell region as well as using a valence deformation density of double-ζ quality are the salient prerequisites to accurately model the electron density of α-boron, and to avoid *core asphericity shifts*. 

## 2. Results and Discussion

### 2.1. QTAIM-Analysis and HCM Refinements of Experimental Structure Factors

**Figure 2:**
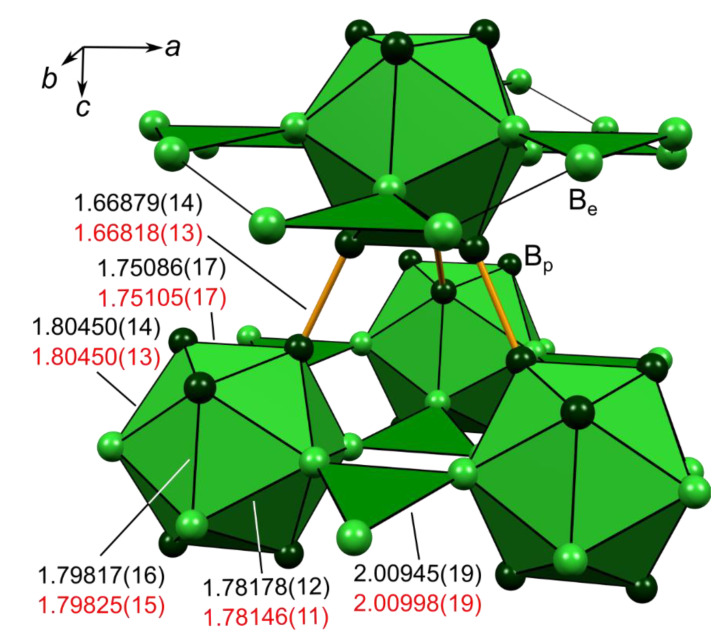
Structural fragment of α-boron, where polar and equatorial boron atoms B_p_ and B_e_ are shown in darker and lighter color, respectively. Apart from delocalized intraicosahedral bonding in the B_12_ icosahedra, (2c,2e)-B_p_-B_p_ bonds are shown as orange sticks and (3c,2e)-B_e_-B_e_-B_e_ bonds are shown as green triangles. Bond lengths are specified in Ångström based on the HCM(*F*_exp_) (black numbers, top) and the final aspherical EHCM(*F*_exp_) (red numbers, bottom) refinement presented in this study.

The rhombohedral structure of α-boron was initially reported by Decker and Kasper and described as a slightly deformed cubic-close-packing of B_12_ icosahedra [54]. The asymmetric unit features polar atoms B_p_, which form short and strong (2c,2e) exo-bonds with neighboring icosahedra, as well as equatorial atoms B_e_ forming somewhat weaker (3c,2e) exo-bonds connecting three icosahedra, see Figure 2. The lengths of the B_p_-B_p_ and B_e_-B_e_ exo-bonds (1.66879(14) & 2.00945(19) Å, as obtained from the HCM refinement of experimental structure factors *F*_exp_) are in very good agreement with the single crystal studies of Mondal et al. (1.6733(5) & 2.0144(3) Å) as well as the synchrotron powder MEM-studies of Nishibori et al. (1.6676(4) & 2.0105(4) Å) [45,49].

**Figure 3:**
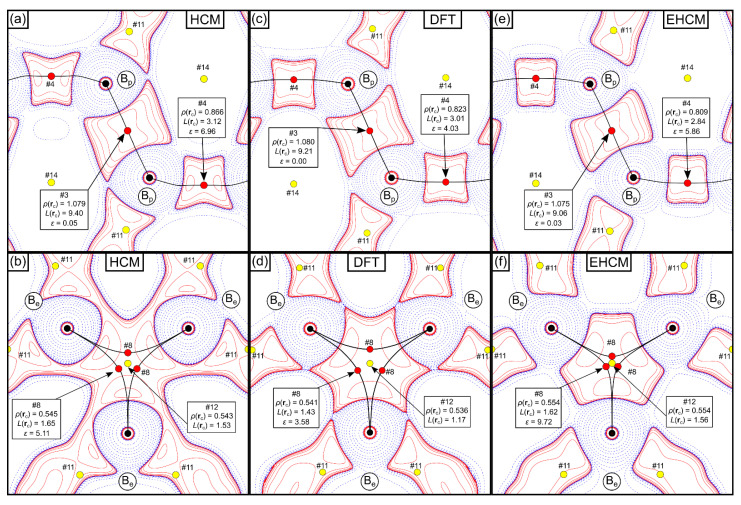
*L*(**r**) = −∇^²^*ρ*(**r**) maps of the intericosahedral (**a**,**c**,**e**) (2c,2e)-B_p_-B_p_ bond and (**b**,**d**,**f**) (3c,2e)-B_e_-B_e_-B_e_ bond in α-boron from the experimental HCM refinement (**a**,**b**), DFT calculation (**c**,**d**) and experimental EHCM(asph) refinement (**e**,**f**). Positive (solid red) and negative (dashed blue) contour lines are shown at ±2·10*^n^*, ±4·10*^n^*, ±8·10*^n^* eÅ^−5^, *n* = ±3, ±2, ±1, 0. *ρ*(**r**_c_)- and *L*(**r**_c_)-values are given in eÅ^−3^ and eÅ^−5^, respectively. The numbers (e.g., #4, #8, #12, etc.) next to the critical points correspond to the numbering scheme of the CPs in Table 1 and Table S1, Supporting Information.

The QTAIM analysis reveals a total of 17 critical points (2 nuclear attractors (NAs), 6 BCPs, 6 RCPs and 3 cage-critical points (CCPs)), for both the experimental (E)HCM refinements, as well as the FP-LAPW DFT calculation. Selected BCPs and RCPs are given in Table 1, for a list of all CPs see Table S1, Supporting Information. Considering the multiplicity of the respective critical points, the Morse rule (*n*(NNA) − *n*(BCP) + *n*(RCP) − *n*(CCP) = 0) is fulfilled [55]. In the following, values derived from the HCM(*F*_exp_) are compared to values derived from the DFT calculation (given in square brackets). The (2c,2e)-B_p_-B_p_ bonds are characterized by the highest *ρ*(**r**_c_)-values of 1.079 [1.080] eÅ^−3^ and the highest *L*(**r**_c_)-values of 9.40 [9.21] eÅ^−5^ of all BCPs in the compound, and a straight bond path in combination with a low bond ellipticity of 0.05 [0.00] (see Figure 3a,c).

In contrast, the (3c,2e)-B_e_-B_e_-B_e_ bond consists of three bond paths which are strongly bent inwards, a characteristic feature of electron deficient bonding [56]. The three BCPs are shifted towards the center of the equilateral triangle, where an RCP is found (see Figure 3b,d). Consequently, the *ρ*(**r**_c_)-values of 0.545 [0.541] eÅ^−3^ and *L*(**r**_c_)-values of 1.65 [1.43] eÅ^−5^ represent the lowest BCP values in the system, with the RCP showing only slightly lower values (*ρ*(**r**_c_) = 0.543 [0.536] eÅ^−3^ and *L*(**r**_c_) = 1.53 [1.17] eÅ^−5^). The corresponding ellipticity ε of 5.11 [3.58] of the BCP is large, with the major axis being tangent to the plane of the bond, indicating delocalization of *ρ*(**r**) over the surface of the triangle [56]. Our results agree very well with the data published by Mondal et al. who reported *ρ*(**r**_c_) values of 1.104/0.561 eÅ^−3^ and *L*(**r**_c_)-values of 9.57/1.24 eÅ^−5^ for the 2c- and 3c-bond, respectively, and the *ρ*(**r**_c_)/*L*(**r**_c_) values of the RCP (0.557 eÅ^−3^/1.06 eÅ^−5^) [45]. *L*(**r**)-maps of both types of bonds are shown in Figure 3a–d. The QTAIM charges (±0.17 e) for the B_p_/B_e_ atoms are small but somewhat larger than the DFT values (±0.08 e), as well as the results of Mondal et al. (±0.07 e) [45].

### 2.2. EHCM Refinements of Calculated Structure Factors and Resolution Dependence of Core Asphericity Shifts

**Figure 4:**
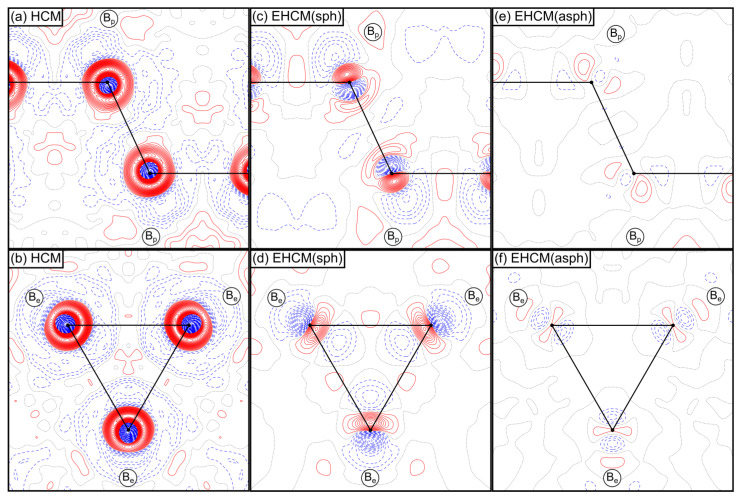
Residual electron density maps of the intericosahedral (**a**,**c**,**e**) (2c,2e)-B_p_-B_p_ bond and (**b**,**d**,**f**) (3c,2e)-B_e_-B_e_-B_e_ bond in α-boron based on refinements of theoretical static structure factors (*F*_sta_, sin(*θ*)/*λ* ≤ 1.6 Å^−1^) employing (**a,b**) an HCM, (**c**,**d**) an EHCM(sph) and (**e**,**f**) an EHCM(asph). Positive (solid red) and negative (dashed blue) contour values are shown in 0.01 eÅ^−3^ steps and the zero contour lines are drawn as dotted black lines.

In order to develop an appropriate EHCM for α-boron, theoretical static structure factors *F*_sta_ will be used, truncated at the same resolution limit as the experimental dataset (sin(*θ*)/*λ* ≤ 1.6 Å^−1^). The necessity to employ an EHCM is already evident from HCM refinements of these structure factors, which results in large residual densities as shown in Figure 4a,b. These residuals are of predominantly spherical character due to the frozen core Ansatz of the HCM, but also show aspherical (dipolar) contributions, as is evident from the displacement of the centers of these residual density distributions from the atomic positions. In the following, we will therefore develop the EHCM in a two-step procedure, i.e., by (*i*) improving the radial (spherical) flexibility and (*ii*) introducing aspherical flexibility in the core region.

For first-row elements, Zhurov and Pinkerton proposed the *P*_00_-method as an alternative to optimized RDFs as used in our previous study on diamond [57]. This method combines the simultaneous refinement of the population parameter *P*_00_ of the single-*ζ* deformation density monopole and the population parameter *P*_v_ of the spherical multiple-ζ valence density *ρ*_v_(*r*). Since the monopole of the deformation density does not contribute significantly to the core region but rather represents parts of *ρ*_v_(*r*) in the valence region, the simultaneous refinement of both parameters allows essentially for a scaling of the inner part of the 2s valence electron density in the core region (for a graphical demonstration, see Figure S5, Supporting Information). However, this only works well if aspherical core polarizations are absent or only weakly pronounced.

In the first step of our analysis, this latter approach has been combined with the refinement of the core population parameter *P*_c_ as well as the core contraction/expansion parameter *κ*_c_. Figure 4c,d shows that the resulting spherical EHCM, termed EHCM(sph), describing the radial electron density distribution of the boron atoms in α-boron rather well since mostly dipolar contributions in the core region remain. The significant improvement in the quality of the model is also highlighted by a drop of the *R*_1_-value from 0.70% in the HCM down to 0.27%, as well as a drop of the magnitude of residual density maxima and minima from +0.246/−0.399 eÅ^−3^ down to +0.149/−0.155 eÅ^−3^. The highest correlation coefficient of 96.8% occurs between the *P*_v_ and *P*_00_ parameters. The increase of values of the *P*_00_ parameters (starting value: 0.0) is 1.022 for B_p_ atoms and 1.427 for B_e_ atoms and thus the reduction of the *P*_v_ parameter shows that the electron density in the core region is depleted with respect to a neutral boron atom (see Figure S5). In addition, the refined core shell parameters *P*_c_ and *κ*_c_ for B_p_ (2.006 and 0.997) and B_e_ (2.008 and 0.998) do not deviate much from their starting value (2.0 and 1.0), and their product *κ*_c_³ *P*_c_ (1.988 for B_p_ and 1.996 for B_e_) also signals a slight depletion of the electron density in the core region. Note that the improvement of the EHCM(sph) originates predominantly from the refinement of the *P*_00_-parameter. Therefore, the parameters *P*_c_ and *κ*_c_ in the corresponding refinements of the experimental data will be kept fixed to the values obtained from the refinement of theoretical structure factors. The magnitude of the electron density depletion in the core region therefore depends on all parameters *P*_v_, *P*_00_, *κ*_v,_ *P*_c_, *κ*_c_ as well as the charge transfer from B_p_ to B_e_ atoms, which, however, is low and does not obscure the trend in case of α-boron.

In the second step *l* = 1 multipoles are introduced for the core shell in the aspherical EHCM, termed EHCM(asph), in order to take the dipolar core polarization into account. Note that for the B_p_ atoms the positive region of the core dipole contribution is opposite to the midpoint of the (2c,2e) bond (Figure 4c), while for the B_e_ atoms the positive region points towards the center of the (3c,2e) bond (Figure 4d), which is the fundamental difference between these two kinds of bonds with respect to the core polarization. While the refinement of such *l* = 1 multipoles was stable for B_p_ atoms, the *κ*_c_’ parameter of the B_e_ atom always decreased significantly as the core dipole contribution remained. This problem could be solved by elevating the description of the valence deformation density of the B_e_ atom to double-ζ quality (the valence deformation density is now composed of two sets “v1” and “v2” of multipole parameters, see Equation (A3) in Appendix A), reminiscent of the Ansatz by Volkov and Coppens [58]. The corresponding residual density maps of the final EHCM(asph) are shown in Figure 4e,f, which are essentially flat and featureless (+0.027/−0.038 eÅ^−^^3^). This improvement of the model is again accompanied by a drop of *R*_1_ down to 0.09%. While the radial maxima of the core deformation density are close to the respective atom at distances of 0.100 Å for B_p_ and 0.113 Å for B_e_ atoms, the corresponding values for the valence density (B_p_: 0.430 Å (v1), B_e_: 0.523 Å (v1) and 0.484 Å (v2)) are located significantly further outwards. The ζ∙κ’ values are 10.60 au^−1^ (core) and 2.46 au^−1^ (v1) for B_p_, as well as 9.35 au^−1^ (core), 2.02 au^−1^ (v1) and 3.28 au^−1^ (v2) for B_e_ atoms. Still, the majority of the bonding electron density around B_e_ atoms is described mostly by the v1 deformation density set, while the v2 set is more localized due to its higher ζ∙κ’ value relative to the v1 parameter set. The smaller value of these distances for B_p_ atoms originates from the description of the localized (2c,2e) bond, while for B_e_ atoms the (3c,2e) multicenter bonds are more delocalized. The correlation coefficient between *P*_00_ and *P*_v_ is further reduced to 92.5%, while the correlations between individual parameters of the v1 and v2 set approach values up to 98.5%, which renders a unique and independent refinement of these parameters in case of experimental data unlikely. As a validation of the model, we note that both the QTAIM charges (+/−0.07 e), as well as the electron density properties at the CPs (Table S2, Supporting Information) agree well with the values based on the original DFT wavefunction. The EHCM(asph) will be our default model in the following analysis.

**Figure 5:**
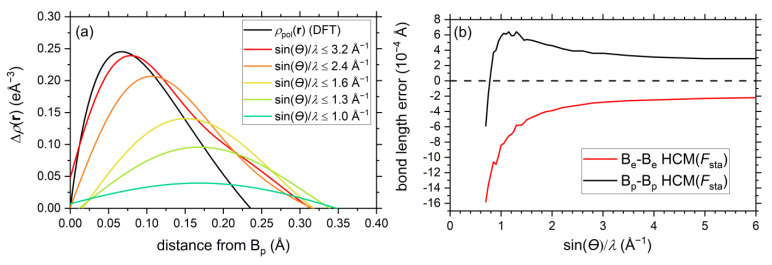
(**a**) EHCM(sph) residual electron density in the proximity of the B_p_ atom along the direction opposite to the intericosahedral B_p_-B_p_-bond as a function of the resolution sin(*θ*)/*λ*. The dipolar contribution *ρ*_pol_(**r**) to the electron density from DFT calculations is shown as a reference and its definition is given in Appendix B. (**b**) Resolution dependence of *core asphericity shifts* of the *exo*-B_p_-B_p_- and *exo*-B_e_-B_e_-bond in refinements of an HCM (with free scale and *U*_iso_ parameters). A horizontal dashed line has been drawn at a value of zero, where the atomic positions correspond to the values used in the DFT calculations.

In order to examine the resolution dependence of the dipolar core polarization, 1D profiles of the residual electron density peak close to the B_p_ position have been plotted for different data resolutions in Figure 5a. Starting from sin(*θ*)/*λ* ≤ 1.0 Å^−1^, the profile maximum is initially very shallow. Upon increasing the data resolution, the Δ*ρ*(**r**) maximum increases and moves closer to the corresponding maximum value of *ρ*_pol_(**r**), which is the dipolar contribution extracted directly from the DFT wavefunction. At the same time, the Δ*ρ*(**r**) maximum shifts closer to the atomic position, also in better agreement with *ρ*_pol_(**r**). For the experimental resolution of sin(*θ*)/*λ* ≤ 1.6 Å^−1^ Δ*ρ*(**r**) reaches about 50% of the maximum value of *ρ*_pol_(**r**) and almost reproduces *ρ*_pol_(**r**) at resolutions of sin(*θ*)/*λ* ≤ 3.2 Å^−1^. Note that the maximum values of the spherical residual density features of the HCM (Figure 4a,b) close to the atomic positions also show a large resolution dependence. While aspherical core polarizations (with a dipolar component) might be compensated by artificial positional changes, the ignorance of spherical core polarizations might cause false scale factor and/or erroneous temperature factors [10,22].

Up to this point, atomic positions were not optimized in refinements against *F*_sta_, since they are highly correlated with multipolar parameters, as suggested by Hirshfeld [19]. Indeed, similar residual density maps can be generated, when either (*i*) the core dipole parameters P_11+_/P_11−_ or (*ii*) positional parameters of the boron atoms are refined, see Figure S1 in the Supporting Information. However, if we employ the HCM, which does not take dipolar core polarization into account, the core asphericity shifts can be determined by refining positional parameters against *F*_sta_ data, see Figure 5b. Note that the same procedure can in principle also be based on the EHCM(sph), but this more flexible model does not allow refinements down to low resolutions.

Due to the different orientations of the dipolar core polarizations, the bond length errors for B_e_ and B_p_ are of opposite sign in the high-resolution limit. The positive value for the *exo*-B_p_-B_p_ bond signals that the bond is about 0.0003 Å too long if core asphericity shifts (backward polarization) are ignored in the refinement model, while the *exo*-B_e_-B_e_ bond (negative value, forward polarization) are falling too short by 0.0002 Å. With decreasing resolution, the absolute values for both errors increase slowly, since the residual density maximum close to the atomic position shifts further away. For the *exo*-B_p_-B_p_ bond a maximum of 0.0006 Å is observed at around sin(*θ*)/*λ* = 1.1–1.2 Å^−1^, while the absolute error keeps on growing for the *exo*-B_e_-B_e_ bond. At low resolutions the core asphericity shifts vanish (Figure 5a) and other shortcomings of the multipolar model (in the valence region) start to dominate, leading to lower bond length values in both cases.

### 2.3. Experimental EHCM Refinements and Correction of Core Asphericity Shifts

In the final step of our analysis we replaced the static theoretical structure factors *F*_sta_ by experimental structure factors *F*_exp_ based on the high-resolution diffraction data (sin(*θ*_max_)/*λ* = 1.579 Å^−1^) at 90 K. To reduce the correlation between individual core parameters (see above) in the EHCM(*F*_exp_) the parameters accounting for the core pseudo atoms for both boron atoms and the “v2” valence deformation density of the B_e_ atom were taken from the corresponding theoretical model EHCM(*F*_sta_). We note that the resolution of the final EHCM(*F*_sta_) was adopted to the resolution of EHCM(*F*_exp_) model. Also, the refinements of experimental structure factors showed a significant sensitivity for core polarization effects. Accordingly, the experimental aspherical EHCM yields a significantly lower R_1_-value (0.90%) than the corresponding spherical EHCM (0.97%) and the classical HCM (1.09%) lacking any core polarization. However, the residual electron density maxima do not change significantly upon considering core asphericity (see Section 3.2).

**Figure 6:**
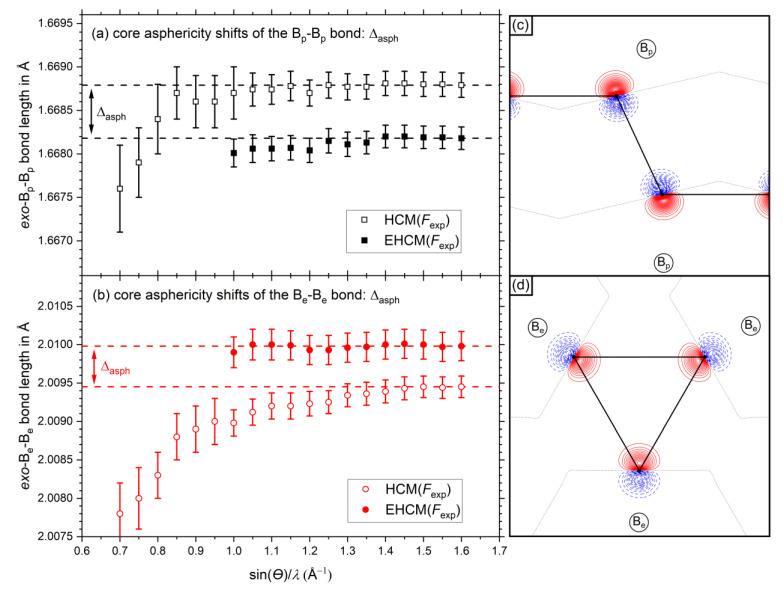
Resolution dependent absolute bond length values of the (**a**) *exo*-B_p_-B_p_-bond and (**b**) *exo*-B_e_-B_e_-bond. Values of the HCM are shown with open symbols, values of the aspherical EHCM(*F*_exp_) are shown with closed symbols. The dashed horizontal lines show the values at maximum resolution as a guide to the eye. The dipolar core polarization contributions according to the EHCM(*F*_sta_) are shown in (**c**) for B_p_ atoms and in (**d**) for B_e_ atoms. Positive (solid red) and negative (dashed blue) contour values are shown in 0.01 eÅ^−3^ steps and the zero contour lines are drawn as dotted black lines.

The experimental aspherical EHCM (full symbols in Figure 6a,b explicitly includes dipolar core polarization and thus minimizes core asphericity shifts. Accordingly, the bond distances between the HCM and the EHCM model differ significantly. These bond length distances can therefore be considered as core asphericity shifts based on experimental data. At the maximum resolution of sin(*θ*)/*λ* ≤ 1.6 Å^−1^ the neglection of core asphericity leads to an increase of the *exo*-B_p_-B_p_-bond by Δ_asph_ = 0.00061 Å and a shortening of the *exo*-B_e_-B_e_-bond by Δ_asph_ = 0.00053 Å, with the corresponding values Δ_asph_ = 0.00053 Å and Δ_asph_ = 0.00048 Å from refinements of *F*_sta_ being slightly lower, respectively (see also Figure S2, Supporting Information). We may conclude that information about dipolar core polarization is indeed contained in the experimental data and leads to core asphericity shifts if not properly treated by the model. These EHCM refinements could be converged to resolutions as low as sin(*θ*)/*λ* ≤ 1.0 Å^−1^ and the bond length values remain rather constant. At even lower resolutions the refinement became unstable due to *P*_v_–*P*_00_ correlations. For further details on core asphericity shifts, see Supporting Information File S1 including Figures S2 and S3.

**Figure 7:**
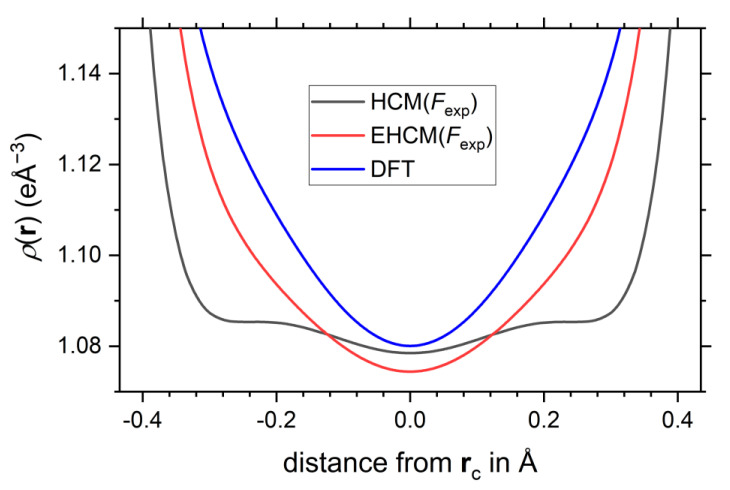
1D-profile of the electron density along the *exo*-B_p_-B_p_ bond.

Note that the properties of the electron density at the critical points change only very little upon application of the aspherical EHCM(*F*_exp_) and remain in good agreement with the DFT calculation, see Figure 3c–f, Table 1 and Table S1 of the Supporting Information. This has already been observed for the EHCM(*F*_sta_) refinements, see Table S2 in the Supporting Information. The QTAIM charges remain at a similar value (±0.20 e) for the B_p_/B_e_). The results for the intermediate spherical EHCM(*F*_exp_) are shown in Table S1 and Figure S4. A mere correction of the atomic coordinates by taking into account core asphericity shifts leaves the CP properties from HCM refinements virtually unaltered. Note that the reduced R-values of the EHCM originate to a large extent from the introduction of the *P*_00_ parameter, which improves the radial electron density distribution of the boron atoms, as well as the double-ζ flexibility introduced for the B_e_ atom. Using the Laplacian of the electron density the subtle changes with regard to the HCM are noticeable mostly in the regions intermediate to the BCP(s) and atomic positions, see Figure 3. Most noteworthy, the 1D-profile of the *exo*-B_p_-B_p_ bond, which is very shallow around the BCP and is prone to the formation of spurious non-nuclear attractors (NNA), improved significantly and follows closer its DFT prediction, see Figure 7, which also goes along with an increase of the curvature λ_3_ from 0.69 eÅ^−5^ in the HCM up to 1.18 eÅ^−5^ in the EHCM(*F*_exp_).

## 3. Materials and Methods

### 3.1. Synthesis

Single crystals of α-boron were grown from Pt-B mixtures at pressures of 5.5(5) GPa and temperatures of 1000 °C in a 6–8 Walker-type multi-anvil device with a 18/12 cell assembly developed by Stoyanov et al. [59]. The sample composition of Pt_15_B_85_ was chosen according to Parakhonskiy et al. [60]. Approximately 70 mg boron powder (99.95%, ChemPur) were placed between two Pt-discs of ca. 110 mg in a boron nitride (*h*-BN) capsule in an Ar-filled glovebox. After reaching the target pressure, the sample was heated to the target temperature within 10 min, dwelling for another 15 min before the sample was quenched by turning off the heater (initial cooling rate approx. −50 °C/s). Amber-colored α-boron crystals embedded in a PtB*_x_*-matrix were recovered by dissolving the coarsely ground sample in hot aqua regia.

### 3.2. Charge Density Study

**Data collection:** An amber-colored single crystal with the dimensions 75 × 139 × 140 µm^3^ was mounted on a MiTeGen MicroMount with small amounts of perfluoropolyalkylether. Data were collected on a Bruker SMART-APEX diffractometer equipped with a D8 goniometer, a microfocus X-ray tube with Ag-Ka radiation (*λ* = 0.56087 Å) and Helios mirror optics. The crystal was cooled to *T* = 90(2) K using an open-flow N_2_-cooling device. A 100 µm thick Al-disc was used to significantly reduce the parasitic radiation contamination with a wavelength of about 3*λ* [61]. A total of 21 ω-scans (180° rotation) were collected at 2*θ*-offsets 0°(8×), −34°(5×), −68°(4×) and −90°(4×) at a detector distance of 4 cm and with ω increments of 0.5°. The exposure times ranged from 15 s to 150 s.

**Data reduction:** Crystal data for α-B (*M* = 10.81 g/mol): trigonal, space group *R*-3*m* (no. 166), *a* = 4.9085(2) Å, *c* = 12.5697(5) Å, *V* = 262.27(2) Å^3^, *Z* = 36, *T* = 90(2) K, *μ* = 0.066 mm^−1^, *D*_calc_ = 2.464 g/cm^3^, *F*_000_ = 180, 23,888 reflections measured (7.676° ≤ 2*θ* ≤ 124.7°, *d*_min_ = 0.317 Å, sin(*θ*_max_)/*λ* = 1.579 Å^−1^, −15 ≤ *h* ≤ 13, −15 ≤ *k* ≤ 15, −38 ≤ *l* ≤ 39), 1093 unique (*R*_int_ = 0.0304, *R*_sigma_ = 0.0098) which were used in all calculations. A numerical absorption correction (*T*_min_ = 0.9685, *T*_max_ = 1.0000) was done using SADABS (Version 2014/2), while the phosphor efficiency was refined to 0.753 [62]. The dataset is complete to *d* > 0.33 Å and 99.2% complete for *d*_min_. The average redundancy/{*I*/*σ*(*I*)} is 21.68/{57.14} and ranges from 81/{168} for the inner shells to 5/{16.1} for the outer most resolution shell, respectively.

**Multipolar refinement**: The initial IAM refinement with anisotropic atomic displacement parameters was carried out using SHELXL and converged to *R*_1_ = 1.78%, w*R*_2_ = 5.46% and GooF = 1.166 for 986 unique reflections (*I* > 2σ(*I*)) and *R*_1_ = 2.06%, w*R*_2_ = 5.59% for all data [63]. The minimum and maximum residual electron density was +0.46/−0.41 eÅ^−^³ and the refinement of an extinction model (SHELX) did not yield any improvement.

All refinements using a multipolar model (HCM or EHCM) have been performed with JANA2006 (Version 25.10.2015), and atomic electron densities were constructed using the Volkov and Macchi atomic wave functions expanded over Slater-type basis functions [64]. The ground state valence configuration (*s^2^p*) for boron atoms was kept throughout all refinements. The equations for these multipolar models are given in Appendix A. The local coordinate system was z ‖ [0,1,0], *y* ‖ [0,0,1] for B_p_ atoms and z ‖ [1,0,0], y ‖ [0,0,1] Be atoms. The refinement of the *l*-dependent multipolar functions 1 ≤ *l* ≤ 4 was carried out in a stepwise manner and finally the valence expansion/contraction parameters *κ*_v_ and *κ*_v_’ were refined for both atoms of the asymmetric unit and converged at *R*_1_ = 1.13% and *wR*_1_ = 1.37% with a residual electron density of +0.15/−0.24 eÅ^−3^. *F*_o_-*F*_c_ plots revealed some of the very strong reflections being overestimated by the model, hinting at small extinction effects. The best results could be obtained via the refinement of an Type-II isotropic extinction parameter *ρ*_iso_ = 1.3(2), according to Becker and Coppens, where extinction effects due to crystallite size dominate [65,66]. The final HCM model converged at *R*_1_ = 1.09%, *wR*_1_ = 1.34% with a residual electron density of +0.18/−0.14 eÅ^−3^ for 969 reflections with *F*_o_ > 3σ(*F*_o_). The maximum reduction due to extinction was 3.0% for the (021)-reflection.

The EHCM(asph) included the free refinement of *l* = 0 multipoles (parameter *P*_00_) for the valence shell as well as the parameters *P*_c_, *κ*_c_, *κ*_c_′ and *P_lm_* (*l* = 1) for the core shell (c) of both boron atoms. For the B_e_-atoms an additional (v2) pseudo atom had to be introduced, involving *P*_v2,*lm*_ parameters (1 ≤ *l* ≤ 3), as well as an independent *κ*_v2_′ parameter, which effectively promotes the radial flexibility of the deformation density from single-ζ to double-ζ quality. In order to avoid linear dependencies, the set of *n_l_*-values was modified from the standard values (2, 2, 2, 3) to (3, 3, 3, 4) for the second v2 set of valence deformation functions (for *l* = 0, 1, 2, 3, respectively). We note that all parameters with indices “c” and “v2” were taken from the refinements against theoretical static structure factors *F*_sta_. The intermediate EHCM(sph) converged at *R*_1_ = 0.97%, *wR*_1_ = 1.27% (Δ*ρ*(**r**)_max_ = +0.18/−0.13 eÅ^−3^) and the final EHCM(asph) yielded *R*_1_ = 0.90%, *wR*_1_ = 1.21% with a residual electron density of +0.19/−0.12 eÅ^−3^. The largest correlation coefficient was found to be 91.5% involving *P*_00_ and *P*_v_-parameters and the extinction parameter increased to *ρ*_iso_ = 2.3(2). All parameters of the experimental HCM and EHCM models are given in Table S3, Supporting Information.

In order to ensure that these models do no overfit the experimental data, we employed the method of *k*-fold (*k* = 20) cross-validation by calculating *R*_cross_ on *F* for all significant reflections [67]. For the transitions HCM → EHCM(sph) and EHCM(sph) → EHCM(asph) we observe a continuous drop of *R*_cross_ with Δ*R*_cross_ = −0.09% and Δ*R*_cross_ = −0.26%, respectively, signaling that the EHCM models do not introduce any overfitting and instead result in a genuine improvement of the description of the experimental electron density.

For all refinements of both experimental and theoretical structure factors, the ζ-values are ζ = 2.464 au^−1^ for the valence deformation functions “v1” and ζ = 9.414 au^−1^ for valence deformation “v2” as well as the core deformations functions.

For the refinement of theoretical static structure factors *F*_sta_, a factor of 100 was applied to the DFT-derived *F*-values and the hkl-file was imported with the “E-format” option in JANA2006 turned on. The coefficients of anomalous dispersion *f* ’ and *f* ” were set to zero and unit weights were used during the refinements.

### 3.3. DFT Calculations

DFT-calculations were performed using the full-potential linear augmented planewave method as implemented in ELK v.6.3.2 [68]. The structural model used in this calculation was taken from the experimental refinement results using a HCM. A 9×9×9 *k*-point grid was employed for the primitive unit cell yielding 85 irreducible *k*-points, and the basis set was extended to *R*_MT_*Gk*_max_ = 10 and *l*_max_ = 14 for the density, potential, and planewaves, while the reciprocal lattice vector was limited to *G*_max_ = 25 a.u.^−1^. An LAPW+lo+LO basis was used in order to reduce the discontinuities at the muffin-tin (MT) sphere boundaries at a distance of *R*_MT_ = 1.3 a.u. The PBE GGA-functional was used for the SCF calculations [69]. For the integration inside the MT-spheres a fine radial mesh (parameter lradstp = 1) in combination with an increased number of radial points (parameter nrmtscf = 4) was used. The QTAIM analysis was performed using critic2 [70]. Structure factors were calculated with ELK up to a resolution of sin(*θ*)/*λ* ≤ 6.0 Å^−1^ and the *hkl*-indices for the primitive unit cell have been transformed into the conventional unit cell using an appropriate transformation matrix.

## 4. Conclusions

Core deformations in α-boron are mainly of a dipolar type. The experimental observation of these subatomic density features can be accomplished using ultra high-resolution diffraction data (sin(*θ*_max_)/λ > 1.5 Å^−1^) in combination with an EHCM model. Ignorance of core deformation effects will otherwise result in artificial *core asphericity shifts*, which are about two orders of magnitude smaller than classic *asphericity shifts*. However, within the last two decades, data quality as well as X-ray intensities of both lab and synchrotron sources have improved significantly, and data collection of experimental resolutions sin(*θ*_max_)/λ > 1.5 Å^−1^ becomes more and more convenient. At such high resolutions, these shifts can become significant and thus should be taken into account by an EHCM or similar models/methods if utmost precision of the structural model and/or electron density distribution is desired.

## Figures and Tables

**Table 1:** List of selected critical points of the topology of the electron density of α-boron from refinements of experimental data of this study, the results from Mondal et al. [45] as well as the periodic DFT calculation. ^a^ *x*,*y*,*z*; ^b^ −*x*+⅔, −*x*+*y*+⅓, −*z*+⅓; ^c^ −*x*+*y*, −*x*,*z*; ^d^ −*x*, −*x*+*y*, −*z*; ^e^ *y*, *x*, −*z*; ^f^ −*x*+*y*, −*x*+1, *z*; ^g^ −*y*+1, *x*−*y*+1, *z*; ^h^ −*y*, *x*−*y*, *z*; ^i^ *x*−*y*, −*y*+1, −*z*.

Cp #	Study/Model	Rank	*m*	*ρ*(r_c_)(eÅ^−3^)	*L*(r_c_)(eÅ^−5^)	*ε*	λ_3_(eÅ^−3^)	Description
3	HCM	(3,−1)	3	1.079	9.40	0.05	0.69	B_p_^a^-B_p_^b^ (exo)
	EHCM(asph)			1.075	9.06	0.03	1.18	
	Mondal et al.			{1.104}	{9.57}	{-}	{-}	
	DFT			[1.080]	[9.21]	[0.00]	[1.69]	
4	HCM	(3,−1)	6	0.866	3.12	6.96	1.09	B_p_^a^-B_p_^c^ (endo)
	EHCM(asph)			0.809	2.84	5.86	1.00	
	Mondal et al.			{0.820}	{2.26}	{-}	{-}	
	DFT			[0.823]	[3.01]	[4.03]	[1.33]	
5	HCM	(3,−1)	6	0.817	3.02	2.31	1.32	B_e_^a^-B_e_^d^ (endo)
	EHCM(asph)			0.803	3.06	4.52	0.98	
	Mondal et al.			{0.804}	{2.47}	{-}	{-}	
	DFT			[0.796]	[2.87]	[2.70]	[1.57]	
6	HCM	(3,−1)	6	0.756	2.58	4.41	1.01	B_p_^a^-B_e_^e^ (endo)
	EHCM(asph)			0.774	2.81	7.16	0.85	
	Mondal et al.			{0.764}	{1.95}	{-}	{-}	
	DFT			[0.768]	[2.60]	[3.45]	[1.45]	
7	HCM	(3,−1)	12	0.756	1.93	3.93	1.44	B_p_^a^-B_e_^a^ (endo)
	EHCM(asph)			0.774	2.60	8.78	0.77	
	Mondal et al.			{0.745}	{1.39}	{-}	{-}	
	DFT			[0.764]	[2.39]	[3.93]	[1.50]	
8	HCM	(3,−1)	6	0.545	1.65	5.11	1.07	B_e_^a^-B_e_^f^ (exo)
	EHCM(asph)			0.554	1.62	9.72	0.47	
	Mondal et al.			{0.561}	{1.24}	{-}	{-}	
	DFT			[0.541]	[1.43]	[3.58]	[1.18]	
9	HCM	(3,+1)	2	0.863	2.76	-	-	B_p_^a^-B_p_^c^-B_p_^h^
	EHCM(asph)			0.800	2.20			
	Mondal et al.			{0.795}	{1.16}			
	DFT			[0.807]	[2.15]			
12	HCM	(3,+1)	2	0.543	1.53	-	-	B_e_^a^-B_e_^f^-B_e_^g^
	EHCM(asph)			0.554	1.56			
	Mondal et al.			{0.557}	{1.06}			
	DFT			[0.536]	[1.17]			

## Data Availability

Data can be made available upon written request to the corresponding author and with a proper justification.

## Supplementary Materials

The following are available online at https://www.mdpi.com/article/10.3390/molecules26144270/s1, Table S1: Complete list of critical points of α-boron from refinements against experimental data, Table S2: Complete list of critical points of α-boron from refinements against theoretical structure factors, Table S3: Parameters of the individual multipolar models for refinements against experimental (Fexp) and theoretical (Fsta) structure factors. Figure S1: Comparison of residual density maps from refinements of the atomic position and core dipole functions. Figure S2: More detailed version of Figure 6a,b of the manuscript. Figure S3: Relative bond length errors of the exo-bonds. Figure S4: Laplacian maps of the experimental EHCM(sph). Figure S5: Effect of the combined refinement of *P*_v_/*P*_00_ parameters on the electron density in the core region. File S1: Further details of core asphericity shifts.

## Sample Availability

α-boron crystals are available from the authors.

## Appendix A

The Hansen–Coppens multipolar model (HCM)

(A1) $$\rho_{\mathrm{at}}\left(r\right)=P_{c}\rho_{c}\left(r\right)+P_{v}\kappa_{v}^{3}\rho_{v}\left(\kappa_{v},r\right)+\overset{l_{\max}}{\underset{l=0}{\sum }}{\left(\kappa_{v,l}^{\prime }\right)}^{3}R_{l}(\kappa_{v,l}^{\prime },r)\overset{l}{\underset{m=-l}{\sum }}P_{lm}d_{lm}\left(\theta ,\phi \right)$$

is based on the original ideas of a pseudoatom model of Stewart in 1977 [6,71]. The electron density of the pseudoatoms $\rho_{\mathrm{at}}\left(r\right)$ is the sum of the spherical (frozen) core density $\rho_{c}\left(r\right)$ (first term), the spherical valence density $\rho_{v}\left(\kappa_{v},r\right)$ (second term) and the so called deformation density (third term), which is expanded in density functions consisting of a radial part $R_{l}(\kappa_{v,l}^{\prime },r)$ and the density-normalized spherical harmonic functions $d_{lm}\left(\theta ,\phi \right)$. The spherical core and valence densities are taken from ground state DFT calculations of isolated atoms using slater-type orbitals (STO) and basis sets of multiple-ζ quality. The core and valence occupation factors *P*_c_ and *P*_v_ account for the number of electrons of the respective pseudoatom and the occupation parameters *P_lm_* model the atomic deformation density. The screening parameter $\kappa_{v}$ accounts for the expansion ($\kappa_{v}<1)$ or contraction ($\kappa_{v}>1$) of the valence shell by modifying the ζ-exponents of the STOs of $\rho_{v}\left(\kappa_{v},r\right)$. All these parameters are determined by a least square refinement, while *P*_c_ and *P*_00_ are commonly not refined and a single, *l*-independent $\kappa_{v}^{\prime }$ value is used. The nodeless radial part is of single-ζ quality and has the form

(A2) $$R_{l}(\kappa_{l}^{\prime },r)=\frac{\zeta^{n_{l}+3}}{\left(n_{l}+2\right)!}{\left(\kappa_{l}^{\prime }\right)}^{3}{\left(\kappa_{l}^{\prime }r\right)}^{n_{l}}e^{-\kappa_{l}^{\prime }\zeta r}$$

where the integer $n_{l}$ parameters must fulfill the condition $n_{l}\geq l$ and the ζ-exponents are often based on energy-optimized values by Clementi and Raimondi [72].

However, this model is often not satisfactory, especially when applied to theoretical structure factors based on DFT calculations. Extended versions of the HCM (EHCM) also make use of a screening parameter $\kappa_{c}$ for the core-shell and explicitly allow for a charge transfer between core and valence pseudo atoms via the explicit refinement of *P*_c_. The EHCM used for this study of α-boron is given by

(A3) $$\begin{array}{ll} \rho_{\mathrm{at}}\left(r\right) & =P_{c}\kappa_{c}^{3}\rho_{c}\left(\kappa_{c},r\right)+{\left(\kappa_{c}^{\prime }\right)}^{3}R_{1}(\kappa_{c}^{\prime },r)\overset{1}{\underset{m=-1}{\sum }}P_{c,1m}d_{1m}\left(\theta ,\phi \right)+P_{v}\kappa_{v}^{3}\rho_{v}\left(\kappa_{v},r\right)\\ & +\overset{l_{\max}}{\underset{l=0}{\sum }}{\left(\kappa_{v1,l}^{\prime }\right)}^{3}R_{l}(\kappa_{v1,l}^{\prime },r)\overset{l}{\underset{m=-l}{\sum }}P_{v1,lm}d_{lm}\left(\theta ,\phi \right)+\left(\overset{l_{\max}}{\underset{l=0}{\sum }}{\left(\kappa_{v2,l}^{\prime }\right)}^{3}R_{l}(\kappa_{v2,l}^{\prime },r)\overset{l}{\underset{m=-l}{\sum }}P_{v2,lm}d_{lm}\left(\theta ,\phi \right)\right). \end{array}$$

The second term in Equation (A3) describes the dipolar core polarization contributions while the fifth term is a second set of deformation density functions which increases the precision to double-ζ quality (v2, used only for B_e_ atoms), while all other variables retain their original meaning. Such models have been described by Volkov and Coppens already some time ago, but without the parametrization of the core density [58].

## Appendix B

In order to extract the dipolar contributions in the core region the value

(A4) $$\rho_{\mathrm{pol}}\left(r\right)=\frac{\rho_{\mathrm{tot}}\left(r_{\mathrm{at}}-r\right)-\rho_{\mathrm{tot}}\left(r_{\mathrm{at}}+r\right)}{2}$$

has been calculated, which allows for simple comparisons with residual density distributions.
