# Supplementary material for: The Effects of Chemical Bonding at Subatomic Resolution: A Case Study on α-Boron
Source: Molecules. 2021 Jul 14;26(14):4270. doi: 10.3390/molecules26144270 (PMC8303496; doi:10.3390/molecules26144270)
Supplement: Supplementary file 1 [file molecules-26-04270-s001.zip › molecules-1255845-SI.pdf]

SUPPLEMENTARY MATERIALS

The Effects of Chemical Bonding at Subatomic Resolution: A Case Study on α-boron

Andreas Fischer, Georg Eickerling and Wolfgang Scherer

Table S1: Complete list of critical points of the topology of the electron density of α-boron from refinements of experimental data of this study, the results from Mondal *et al.* (ref 45 in the manuscript) as well as the periodic DFT calculation. [a] *x,y,z* [b] *−x+⅔,−x+y+⅓,−z+⅓* [c] *−x+y,−x,z* [d] *−x,−x+y,−z* [e] *y,x,−z* [f] *−x+y,−x+1,z* [g] *−y+1,x−y+1,z* [h] *−y,x−y,z* [i] *x−y,−y+1,−z*

cp #	study	rank	*m*	$\rho(\mathbf{r}_c)$ (eÅ^{-3})	$L(\mathbf{r}_c)$ (eÅ^{-5})	ε	λ_3 (eÅ^{-5})	location description
1	-	(3,−3)	6	-	-	-	-	B_p^a
2	-	(3,−3)	6	-	-	-	-	B_e^a
3	HCM<br>EHCM(sph)<br>EHCM(asph)<br>Mondal<br>DFT	(3,−1)	3	1.079<br>1.043<br>1.075<br>{1.104}<br>[1.080]	9.40<br>8.84<br>9.06<br>{9.57}<br>[9.21]	0.05<br>0.02<br>0.03<br>{-}<br>[0.00]	0.69<br>0.76<br>1.18<br>{-}<br>[1.69]	B_p^a-B_p^b (exo)
4	HCM<br>EHCM(sph)<br>EHCM(asph)<br>Mondal<br>DFT	(3,−1)	6	0.866<br>0.829<br>0.809<br>{0.820}<br>[0.823]	3.12<br>2.90<br>2.84<br>{2.26}<br>[3.01]	6.96<br>5.90<br>5.86<br>{-}<br>[4.03]	1.09<br>1.09<br>1.00<br>{-}<br>[1.33]	B_p^a-B_p^c (endo)
5	HCM<br>EHCM(sph)<br>EHCM(asph)<br>Mondal<br>DFT	(3,−1)	6	0.817<br>0.791<br>0.803<br>{0.804}<br>[0.796]	3.02<br>2.88<br>3.06<br>{2.47}<br>[2.87]	2.31<br>3.14<br>4.52<br>{-}<br>[2.70]	1.32<br>1.12<br>0.98<br>{-}<br>[1.57]	B_e^a-B_e^d (endo)
6	HCM<br>EHCM(sph)<br>EHCM(asph)<br>Mondal<br>DFT	(3,−1)	6	0.756<br>0.748<br>0.774<br>{0.764}<br>[0.768]	2.58<br>2.55<br>2.81<br>{1.95}<br>[2.60]	4.41<br>5.82<br>7.16<br>{-}<br>[3.45]	1.01<br>1.02<br>0.85<br>{-}<br>[1.45]	B_p^a-B_e^e (endo)
7	HCM<br>EHCM(sph)<br>EHCM(asph)<br>Mondal<br>DFT	(3,−1)	12	0.756<br>0.743<br>0.774<br>{0.745}<br>[0.764]	1.93<br>1.94<br>2.60<br>{1.39}<br>[2.39]	3.93<br>5.46<br>8.78<br>{-}<br>[3.93]	1.44<br>1.29<br>0.77<br>{-}<br>[1.50]	B_p^a-B_e^a (endo)
8	HCM<br>EHCM(sph)<br>EHCM(asph)<br>Mondal<br>DFT	(3,−1)	6	0.545<br>0.541<br>0.554<br>{0.561}<br>[0.541]	1.65<br>1.50<br>1.62<br>{1.24}<br>[1.43]	5.11<br>4.36<br>9.72<br>{-}<br>[3.58]	1.07<br>1.03<br>0.47<br>{-}<br>[1.18]	B_e^a-B_e^f (exo)
9	HCM<br>EHCM(sph)<br>EHCM(asph)<br>Mondal<br>DFT	(3,+1)	2	0.863<br>0.823<br>0.800<br>{0.795}<br>[0.807]	2.76<br>2.40<br>2.20<br>{1.15}<br>[2.15]	-	-	B_p^a-B_p^c-B_p^h
10	HCM<br>EHCM(sph)<br>EHCM(asph)<br>Mondal	(3,+1)	6	0.731<br>0.722<br>0.759<br>{0.704}	0.67<br>0.94<br>1.76<br>{1.96}	-	-	B_p^a-B_p^h-B_e^a

	DFT			[0.732]	[1.14]			
11	HCM<br>EHCM(sph)<br>EHCM(asph)<br>Mondal<br>DFT	(3,+1)	12	0.727<br>0.723<br>0.754<br>{0.716}<br>[0.728]	0.98<br>1.32<br>1.88<br>{4.32}<br>[1.19]	-	-	B_p^a-B_e^a-B_e^e
12	HCM<br>EHCM(sph)<br>EHCM(asph)<br>Mondal<br>DFT	(3,+1)	2	0.543<br>0.538<br>0.554<br>{0.557}<br>[0.536]	1.53<br>1.31<br>1.56<br>{1.06}<br>[1.17]	-	-	B_e^a-B_e^f-B_e^g
13	HCM<br>EHCM(sph)<br>EHCM(asph)<br>Mondal<br>DFT	(3,+1)	3	0.277<br>0.296<br>0.276<br>{0.239}<br>[0.259]	−2.02<br>-1.74<br>-1.73<br>{−2.10}<br>[−1.88]	-	-	B_e^a-B_e^d-B_e^f-B_e^i
14	HCM<br>EHCM(sph)<br>EHCM(asph)<br>DFT	(3,+1)	6	0.102<br>0.110<br>0.097<br>[0.088]	−1.10<br>-1.07<br>-1.14<br>[−1.05]	-	-	side surface of tetrahedrons formed by B_{12}-icosahedra
15	HCM<br>EHCM(sph)<br>EHCM(asph)<br>DFT	(3,+3)	1	0.079<br>0.090<br>0.085<br>[0.116]	−2.71<br>-2.62<br>-2.97<br>[−2.05]	-	-	center of B_{12} icosahedron
16	HCM<br>EHCM(sph)<br>EHCM(asph)<br>DFT	(3,+3)	2	0.059<br>0.065<br>0.061<br>[0.059]	−0.97<br>-0.93<br>-0.97<br>[−0.83]	-	-	B_{12} tetrahedral void
17	HCM<br>EHCM(sph)<br>EHCM(asph)<br>DFT	(3,+3)	1	0.028<br>0.032<br>0.031<br>[0.021]	−0.31<br>-0.34<br>-0.29<br>[−0.26]	-	-	B_{12} octahedral void

Table S2: Complete list of critical points of the electron density of α-boron as obtained from the refinements of theoretical structure factors F_{sta}.

cp #	study	rank	m	$\rho(\mathbf{r}_c)$ (eÅ^{-3})	$L(\mathbf{r}_c)$ (eÅ^{-5})	ε	λ_3 (eÅ^{-5})	location description
1	-	(3,−3)	6	-	-	-		B_p^a
2	-	(3,−3)	6	-	-	-		B_e^a
3	HCM	(3,−1)	3	1.095	9.18	0.02	1.32	B_p^a-B_p^b (exo)
	EHCM(sph)			1.082	9.11	0.00	1.42	
	EHCM(asph)			1.073	8.40	0.01	2.15	
	DFT			1.080	9.21	0.00	1.69	
4	HCM	(3,−1)	6	0.827	2.31	2.70	2.15	B_p^a-B_p^c (endo)
	EHCM(sph)			0.805	2.09	3.15	2.04	
	EHCM(asph)			0.815	2.40	3.64	1.91	
	DFT			0.823	3.01	4.03	1.33	
5	HCM	(3,−1)	6	0.798	2.06	1.87	2.47	B_e^a-B_e^d (endo)
	EHCM(sph)			0.783	2.11	2.69	2.08	
	EHCM(asph)			0.792	2.46	2.92	1.81	
	DFT			0.796	2.87	2.70	1.57	
6	HCM	(3,−1)	6	0.760	1.60	2.53	2.35	B_p^a-B_e^e (endo)
	EHCM(sph)			0.749	1.63	3.38	2.06	
	EHCM(asph)			0.759	2.00	4.17	1.78	
	DFT			0.768	2.60	3.45	1.45	
7	HCM	(3,−1)	12	0.750	1.16	3.34	2.56	B_p^a-B_e^a (endo)
	EHCM(sph)			0.749	1.57	3.83	2.15	
	EHCM(asph)			0.758	1.93	4.22	1.72	
	DFT			0.764	2.39	3.93	1.50	
8	HCM	(3,−1)	6	0.529	0.73	2.09	2.12	B_e^a-B_e^f (exo)
	EHCM(sph)			0.538	1.09	2.79	1.61	
	EHCM(asph)			0.537	1.22	3.53	1.20	
	DFT			0.541	1.43	3.58	1.18	
9	HCM	(3,+1)	2	0.801	1.15	-	-	B_p^a-B_p^c-B_p^h
	EHCM(sph)			0.783	1.22			
	EHCM(asph)			0.798	1.60			
	DFT			0.807	2.15			
10	HCM	(3,+1)	6	0.711	0.03	-	-	B_p^a-B_p^h-B_e^a
	EHCM(sph)			0.712	0.37			
	EHCM(asph)			0.728	0.88			
	DFT			0.732	1.14			
11	HCM	(3,+1)	12	0.712	0.16	-	-	B_p^a-B_e^a-B_e^e
	EHCM(sph)			0.712	0.56			
	EHCM(asph)			0.725	0.99			
	DFT			0.728	1.19			
12	HCM	(3,+1)	2	0.520	0.50	-	-	B_e^a-B_e^f-B_e^g
	EHCM(sph)			0.533	0.89			
	EHCM(asph)			0.533	1.05			
	DFT			0.536	1.17			
13	HCM	(3,+1)	3	0.253	-2.02	-	-	B_e^a-B_e^d-B_e^f-B_e^i

	EHCM(sph)<br>EHCM(asph)<br>DFT			0.262<br>0.258<br>0.259	-1.91<br>-1.91<br>-1.88			
14	HCM<br>EHCM(sph)<br>EHCM(asph)<br>DFT	(3,+1)	6	0.076<br>0.086<br>0.088<br>0.088	-1.13<br>-1.13<br>-1.09<br>-1.05	-	-	side surface of tetrahedrons formed by B_{12}-icosahedra
15	HCM<br>EHCM(sph)<br>EHCM(asph)<br>DFT	(3,+3)	1	0.065<br>0.086<br>0.095<br>0.116	-2.62<br>-2.47<br>-2.63<br>-2.05	-	-	center of B_{12} icosahedron
16	HCM<br>EHCM(sph)<br>EHCM(asph)<br>DFT	(3,+3)	2	0.041<br>0.054<br>0.055<br>0.059	-0.92<br>-0.90<br>-0.90<br>-0.83	-	-	B_{12} tetrahedral void
17	HCM<br>EHCM(sph)<br>EHCM(asph)<br>DFT	(3,+3)	1	0.019<br>0.022<br>0.021<br>0.021	-0.22<br>-0.27<br>-0.27<br>-0.26	-	-	B_{12} octahedral void

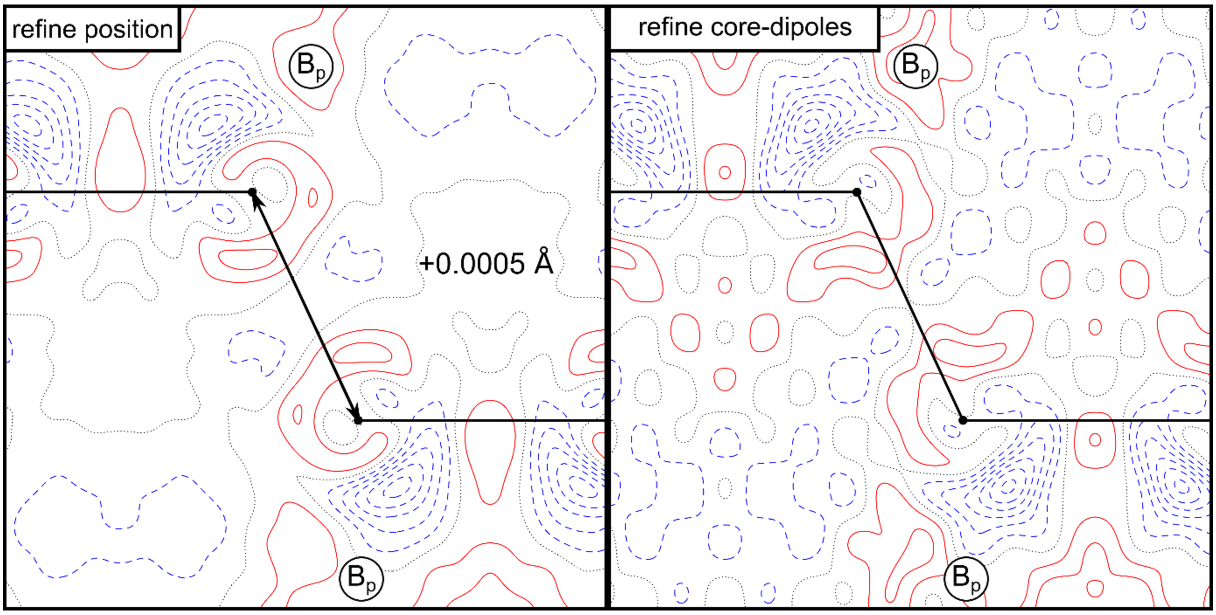

Figure S1: Comparison of residual density maps from refinements of the atomic position (left) and core dipole functions (right) against theoretical structure factors F_{sta}. Positive (solid red) and negative (dashed blue) contour values are shown in 0.01 eÅ^{-3} steps and the zero contour lines are drawn as dotted black lines. Refining the positions leads to a bond elongation of 0.0005 Å as indicated by the double-headed arrow.

Table S3: Parameters of the individual multipolar models for refinements against experimental (F_{exp}) and theoretical (F_{sta}) structure factors. * the „valence v2“ and „core“ atom share the same parameters P_c and κ_c. † value not refined and taken from refinements against F_{sta}.

F_{exp}	HCM	EHCM(sph)	EHCM(asph)
scale	2.392(4)	2.423(6)	2.430(4)
ρ_{iso}	1.25(2)	1.9(3)	2.3(2)
B_p (valence v1)			
occ	1.0	1.0	1.0
x	0.237800(15)	0.237799(15)	0.237825(14)
y	0.118900	0.118900	0.118912
z	0.108678(5)	0.108679(5)	0.108701(4)
U_{11}	0.00273(2)	0.00264(3)	0.00264(3)
U_{22}	0.00310(2)	0.00302(3)	0.00302(3)
U_{33}	0.00263(2)	0.00254(3)	0.00255(3)
U_{12}	0.001362	0.001321	0.001320
U_{13}	-0.00027(1)	-0.000265(10)	-0.000262(10)
U_{23}	-0.000133	-0.000133	-0.000131
U_{eq}	0.002860(14)	0.002777(18)	0.002781(18)
P_v	2.92(4)	1.4(3)	1.1(2)
κ_v	0.935(10)	0.954(19)	0.95(3)
κ_v'	0.91(3)	0.94(4)	0.98(3)
P_{00}	0.000000	1.5(3)	1.4(2)
P_{11+}	0.018(16)	0.020(15)	-0.058(16)
P_{11-}	0.078(16)	0.076(15)	0.144(19)
P_{20}	-0.095(13)	-0.089(12)	-0.078(10)
P_{22+}	-0.027(14)	-0.037(13)	-0.024(11)
P_{22-}	-0.136(12)	-0.127(13)	-0.093(9)
P_{31+}	0.046(13)	0.053(12)	0.059(11)
P_{31-}	-0.141(16)	-0.133(17)	-0.119(13)
P_{33+}	0.20(2)	0.20(3)	0.201(17)
P_{33-}	0.001(12)	0.017(10)	0.024(9)
P_{40}	0.043(15)	0.009(14)	0.009(12)
P_{42+}	0.009(17)	0.009(17)	0.003(14)
P_{42-}	-0.02(3)	-0.00(3)	0.005(16)
P_{44+}	0.031(18)	0.023(17)	0.014(14)
P_{44-}	0.001(13)	-0.007(12)	-0.012(10)
B_p (core)			
occ	1.0	1.0	1.0
P_c	2.000000	2.005752†	2.001875†
κ_c	1.000000	0.996701†	0.999194†
κ_c'	–	–	1.126216†
P_{11+}	–	–	0.001021†
P_{11-}	–	–	-0.003796†

F_{exp}	HCM	EHCM(sph)	EHCM(asph)
		B_e (valence v1)	
occ	1.0	1.0	1.0
x	0.196873	0.196879	0.196837
y	0.393746(16)	0.393758(15)	0.393673(14)
z	0.024302(5)	0.024302(5)	0.024299(5)
U_{11}	0.003159(19)	0.00301(3)	0.00305(2)
U_{22}	0.00287(2)	0.00272(3)	0.00277(3)
U_{33}	0.00316(2)	0.00300(3)	0.00304(3)
U_{12}	0.001435	0.001361	0.001384
U_{13}	-0.000020(5)	-0.000019(5)	-0.000018(5)
U_{23}	-0.000040	-0.000039	-0.000036
U_{eq}	0.003095(14)	0.002944(18)	0.002986(15)
P_v	3.08(4)	0.9(5)	0.9(4)
κ_v	0.926(9)	0.92(4)	1.09(4)
κ_v'	0.85(3)	0.94(3)	0.81(3)
P_{00}	0.000000	2.2(3)	2.56(17)
P_{11+}	-0.18(3)	-0.125(16)	-0.34(4)
P_{11-}	-0.035(14)	-0.018(11)	-0.059(17)
P_{20}	0.034(13)	0.013(10)	-0.132(18)
P_{22+}	-0.022(13)	-0.032(11)	0.21(2)
P_{22-}	-0.032(12)	-0.023(9)	-0.13(3)
P_{31+}	-0.135(19)	-0.107(15)	-0.075(19)
P_{31-}	-0.053(15)	-0.041(12)	-0.09(2)
P_{33+}	0.20(3)	0.174(16)	0.22(3)
P_{33-}	0.094(15)	0.075(11)	0.22(3)
P_{40}	-0.02(3)	-0.025(17)	-0.06(3)
P_{42+}	0.02(3)	0.022(17)	0.07(3)
P_{42-}	0.02(3)	0.02(3)	0.06(3)
P_{44+}	0.07(3)	0.037(16)	-0.01(3)
P_{44-}	-0.050(17)	-0.044(12)	-0.080(19)
		B_e (core)	
occ	1.0	1.0	0.5*
P_c	2.000000	2.007725†	2.011135†
κ_c	1.000000	0.998263†	0.996957†
κ_c'	–	–	0.992718†
P_{11+}	–	–	0.007922†
P_{11-}	–	–	0.001127†
		B_e (valence v2)	
occ	–	–	0.5*
κ_{v2}'	–	–	0.348354†
P_{11+}	–	–	0.111062†
P_{11-}	–	–	0.042466†
P_{20}	–	–	0.143420†
P_{22+}	–	–	-0.247276†
P_{22-}	–	–	0.057953†
P_{31+}	–	–	-0.097147†
P_{31-}	–	–	0.013475†
P_{33+}	–	–	0.057291†
P_{33-}	–	–	-0.029583†

***F*sta**	**HCM**	**EHCM(sph)**	**EHCM(asph)**
scale	98.80968	100.00000	100.00000
B_p (valence v1)			
occ	1.0	1.0	1.0
P_v	2.970676	1.906949	1.798231
U_{iso}	0.000000	0.000000	0.000000
κ_v	0.987113	1.024930	1.008928
κ_v'	0.867116	0.902841	0.999876
P_{00}	0.000000	1.022481	0.879467
P_{11+}	-0.014751	0.004075	-0.037922
P_{11-}	0.033440	0.054917	0.129619
P_{20}	-0.107229	-0.097928	-0.070309
P_{22+}	-0.021802	-0.025685	-0.015897
P_{22-}	-0.145884	-0.139408	-0.080796
P_{31+}	0.075809	0.084118	0.050749
P_{31-}	-0.157719	-0.146018	-0.107901
P_{33+}	0.226153	0.206517	0.175569
P_{33-}	0.047387	0.034883	0.013719
P_{40}	0.004292	0.006547	0.008700
P_{42+}	0.029197	0.002021	0.000324
P_{42-}	0.030556	0.014826	0.005373
P_{44+}	0.012848	-0.000608	0.01757
P_{44-}	0.032987	0.031303	0.006025
B_p (core)			
occ	1.0	1.0	1.0
P_c	2.000000	2.005752	2.001875
κ_c	1.000000	0.996701	0.999194
κ_c'	–	–	1.126216
P_{11+}	–	–	0.001021
P_{11-}	–	–	-0.003796

F_{sta}	HCM	EHCM(sph)	EHCM(asph)
B_e (valence v1)			
occ	1.0	1.0	1.0
P_v	3.029322	1.629217	1.589855
U_{iso}	0.000000	0.000000	0.000000
κ_v	0.994612	1.019481	1.054493
κ_v'	0.912220	0.936793	0.821156
P_{00}	0.000000	1.427873	1.719434
P_{11+}	−0.126289	−0.121895	−0.288148
P_{11-}	−0.010054	−0.028842	−0.062408
P_{20}	0.011378	0.005510	−0.121370
P_{22+}	0.003626	−0.014999	0.185338
P_{22-}	−0.021418	−0.016922	−0.105249
P_{31+}	−0.12756	−0.121687	−0.055604
P_{31-}	−0.019615	−0.018638	−0.068528
P_{33+}	0.171735	0.179353	0.194364
P_{33-}	0.070011	0.079635	0.180315
P_{40}	0.023885	−0.008621	−0.047154
P_{42+}	0.05538	0.028484	0.058562
P_{42-}	0.016002	0.003843	0.041137
P_{44+}	0.023583	0.019310	−0.004083
P_{44-}	−0.013882	−0.014066	−0.057457
B_e (core)			
occ	1.0	1.0	0.5*
P_c	2.000000	2.007725	2.011135
κ_c	1.000000	0.998263	0.996957
κ_c'	–	–	0.992718
P_{11+}	–	–	0.007922
P_{11-}	–	–	0.001127
B_e (valence v2)			
occ	–	–	0.5*
κ_{v2}'	–	–	0.348354
P_{11+}	–	–	0.111062
P_{11-}	–	–	0.042466
P_{20}	–	–	0.143420
P_{22+}	–	–	−0.247276
P_{22-}	–	–	0.057953
P_{31+}	–	–	−0.097147
P_{31-}	–	–	0.013475
P_{33+}	–	–	0.057291
P_{33-}	–	–	−0.029583

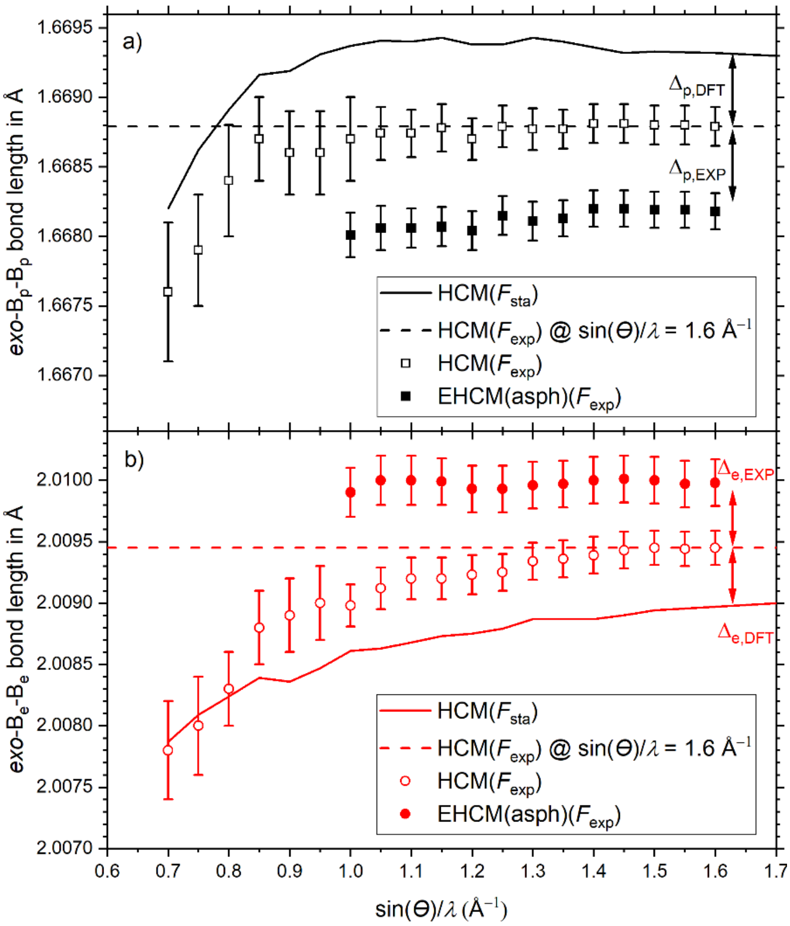

Figure S2. Absolute resolution dependent bond length values of the a) *exo*-B_p-B_p-bond and b) *exo*-B_e-B_e-bond. Solid lines are based on refinements of an HCM against F_{sta} and are taken from Figure 5b. Values of the HCM are shown with open symbols, values of the EHCM(asph) are shown with closed symbols. The dash horizontal lines show the values used for the DFT calculation.

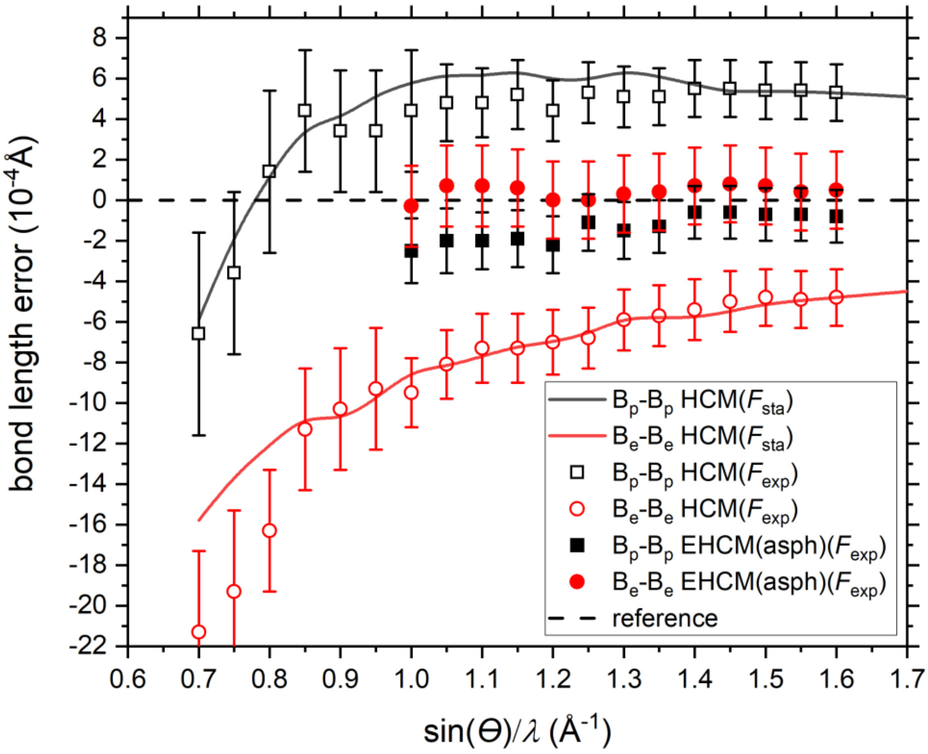

Figure S3. Relative bond length errors for the *exo*-bonds between B_p (black) and B_e (red) atoms. Solid lines are taken from Figure 5b of the manuscript and have been set equal to the HCM(F_{exp}) refinements by translation at the maximum experimental resolution. The dashed horizontal line signals the absence of core asphericity shifts based on the values of $\Delta_{asph,DFT}$.

File S1: Further details of core asphericity shifts

Figure S6 contains also contains the bond length values from HCM(F_{sta}) refinements as solid lines. Note that the DFT calculations (solid lines) are based on the structural model from the experimental HCM refinements (open symbols), which ignored core asphericity. As a consequence, the structural model employed for the DFT calculations is biased by asphericity shifts and is not representing an equilibrium geometry. Therefore, the structure factors F_{sta} derived from these DFT calculations are already biased by asphericity shifts induced by dipolar core polarizations of the experimental data . These polarization effects can be compensated by refinements of an HCM against these theoretical F_{sta} when the coordinates of the two independent boron atoms B_p and B_e are allowed to relax. In that case, the polarization effects are compensated by aspericity shifts of the B_p and B_e positions with regard to the constrained positions in the DFT model. Note that these shifts are of the same extent as the asphercity shifts between the experimental EHCM and HCM. They shift in opposite direction, because dipolar core polarization is now taken into account when the HCM is replaced by an EHCM. This shift appears to be rather independent of the resolution, except for the B_e-B_e bond length below $\sin(\theta)/\lambda \leq 0.9$ Å^{-1}.

Using $\Delta_{asph,EXP} = \Delta_{asph,DFT}$ as a rule, the resulting bond length errors can be plotted on a relative scale by translating all datapoints of each B_p-B_p and B_e-B_e bond so that experimental and theoretical HCM values match at $\sin(\theta)/\lambda = 1.6$ Å^{-1}, see Figure S6. The experimental and theoretical HCM data match within 1 s.u. for the B_p-B_p error in the whole resolution range 0.7 Å$^{-1} \leq \sin(\theta)/\lambda \leq 1.6$ Å^{-1} and down to $\sin(\theta)/\lambda \geq 0.85$ Å^{-1} for the B_e-B_e error value, which highlights the excellent quality of the experimental dataset. This confirms that *core asphericity shifts* can be effectively taken into account using an EHCM in case of α-boron. We also demonstrate that the bond length error in the HCM model is significantly reduced in the EHCM to values below 0.0001 Å. Note that due to the small bond length changes, a DFT calculation based on the EHCM(asph) structural model would produce only minute changes of the valence electron density distribution, which is the origin of dipolar core polarization in the core region of the atoms according to the Hellman-Feynman condition. Therefore, HCM refinements based on static structure factors F_{sta} originating from the EHCM(asph) structural model would yield almost identical values for the *core asphericity shifts*. This renders an iterative approach as used in Hirshfeld atoms refinements (see Introduction of the manuscript) unnecessary.

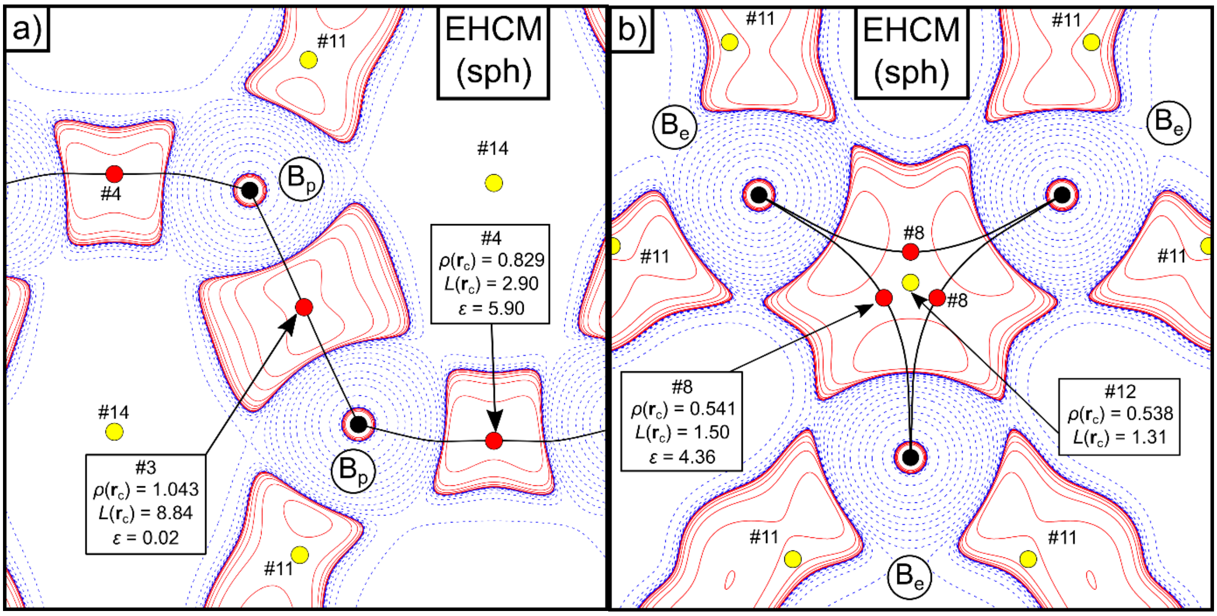

Figure S4: $L(\mathbf{r}) = -\nabla^2\rho(\mathbf{r})$ maps of the intericosahedral (a) (2c,2e)-B_p-B_p bond and (b) (3c,2e)-B_e-B_e-B_e bond in α-boron from the experimental EHCM(sph) refinement. Positive (solid red) and negative (dashed blue) contour lines are shown at $\pm2\cdot10^n$, $\pm4\cdot10^n$, $\pm8\cdot10^n$ eÅ^{-3}, n = ±3, ±2, ±1, 0. $\rho(\mathbf{r}_c)$- and $L(\mathbf{r}_c)$-values are given in eÅ^{-3} and eÅ^{-5}, respectively. The numbers (e.g. #4, #8, #12, etc.) next to the critical points correspond to the numbering scheme of the CPs in Table S1.

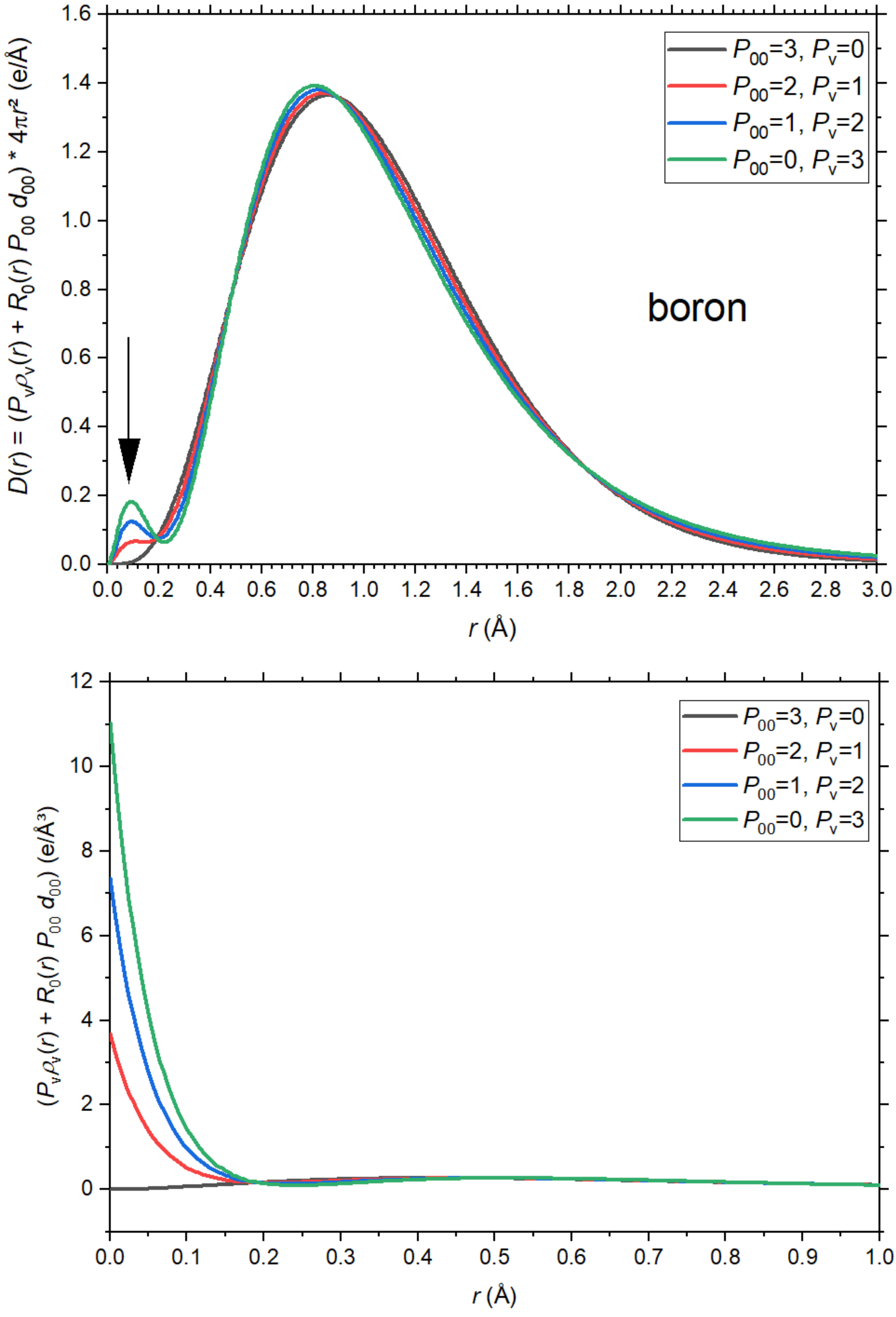

Figure S5: Effect of the combined refinement of P_v/P_{00} parameters of an (E)HCM on the valence electron density in the core region of a neutral, isolated boron atom in its ground state from the Volkov-Macchi database (VM). The scaling effect on the contribution to the core region is highlighted by an arrow. Further parameters of the model are ζ = 2.464 au^{-1}, κ_v = 1.0, κ_v' = 1.0 and n_0 = 2.
